# Development of Extraction Method for Determination of Saponins in Soybean-Based Yoghurt Alternatives: Effect of Sample pH

**DOI:** 10.3390/foods12112164

**Published:** 2023-05-27

**Authors:** Anastassia Bljahhina, Maria Kuhtinskaja, Tiina Kriščiunaite

**Affiliations:** 1 Center of Food and Fermentation Technologies (TFTAK), Mäealuse 2/4, 12618 Tallinn, Estonia; anastassia.bljahhina@tftak.eu; 2 Department of Chemistry and Biotechnology, Tallinn University of Technology, Akadeemia tee 15, 12618 Tallinn, Estonia

**Keywords:** bitterness, *Glycine max*, plant-based foods, plant proteins, LC-MS

## Abstract

The number of plant-based dairy alternative products on the market is growing rapidly. In the case of soybean-based yoghurt alternatives, it is important to trace the content of saponins, the phytomicronutrients with a disputable health effect, which are likely to be responsible for the bitter off-taste of the products. We present a new sample extraction method followed by hydrophilic interaction liquid chromatography with mass spectrometric detection (HILIC-MS) for identifying and quantifying soyasaponins in soybean-based yoghurt alternatives. Soyasaponin Bb, soyasaponin Ba, soyasaponin Aa, and soyasaponin Ab were quantified using commercially available standard compounds and with asperosaponin VI as the internal standard. As the recoveries of soyasaponins were unacceptable in yoghurt alternatives at their natural acidic pH, the adjustment of pH was performed as one of the first steps in the sample extraction procedure to achieve the optimum solubility of soyasaponins. The validation of the method included the assessment of linearity, precision, limit of detection and limit of quantification (LOQ), recovery, and matrix effect. The average concentrations of soyasaponin Bb, soyasaponin Ba, soyasaponin Ab, and soyasaponin Aa in several measured soybean-based yoghurt alternatives utilising the developed method were 12.6 ± 1.2, 3.2 ± 0.7, 6.0 ± 2.4 mg/100 g, and below the LOQ, respectively. This method provides an efficient and relatively simple procedure for extracting soyasaponins from yoghurt alternatives followed by rapid quantification using HILIC-MS and could find a rightful application in the development of healthier and better-tasting dairy alternatives.

## 1. Introduction

In recent decades, the market for plant-based dairy alternatives has vastly expanded. In addition to cereals, pseudocereals, and nuts, legumes are typically used to produce plant-based dairy alternatives. Due to their high protein content and quality, legumes such as soybeans (*Glycine max* L.) are widely used to manufacture dairy alternatives [1]. However, there is still a lack of quantitative data on the migration of phytonutrients during food processing from plant-based protein sources to the final consumable products. Along with macronutrients, soybeans contain several classes of biologically active compounds, including naturally occurring complex oleanane triterpenoid glycoside saponins [2]. Chitisankul et al. studied saponin content in nine soybean varieties and fourteen different soybean-based milk alternatives. The average total soyasaponin content reported was 246 ± 92 and 269 ± 140 μmol per 100 g dry weight basis (dwb), respectively [3,4], suggesting a transfer of saponins from the dry matter throughout the production chain of plant-based milk alternatives.

The dietary preferences of many consumers are shifting towards plant-based products due to environmental, health, and ethical reasons. Thus, from a nutritional point of view, it is important to quantify phytochemicals from emerging plant-based alternatives. Although human cells are not able to degrade saponins [5], some bacteria from gut microbiota convert saponins into sapogenols [6] and enter the bloodstream [7]. Until now, the data on the effects of saponins on human health are controversial. Negative consequences of high saponin consumption have been proven in livestock; e.g., health issues in the digestive tract of ruminants as well as decreases in wool, milk, and egg production were observed [8]. In addition, high concentrations of saponins may lead to the inefficient absorption of fat-soluble vitamins and damage the membrane of the intestinal inner epithelial wall [9]. On the contrary, several in vitro and in vivo studies have shown the positive immunological and antiviral effects of soyasaponins [10]. In addition, anti-cancerogenic [6], hepato-protective [11], anti-inflammatory [12], and anti-obesity effects [13,14] have been reported. The beneficial and deleterious nutritional properties of saponins are likely to be dose- and diet-dependent.

**Figure 1:**
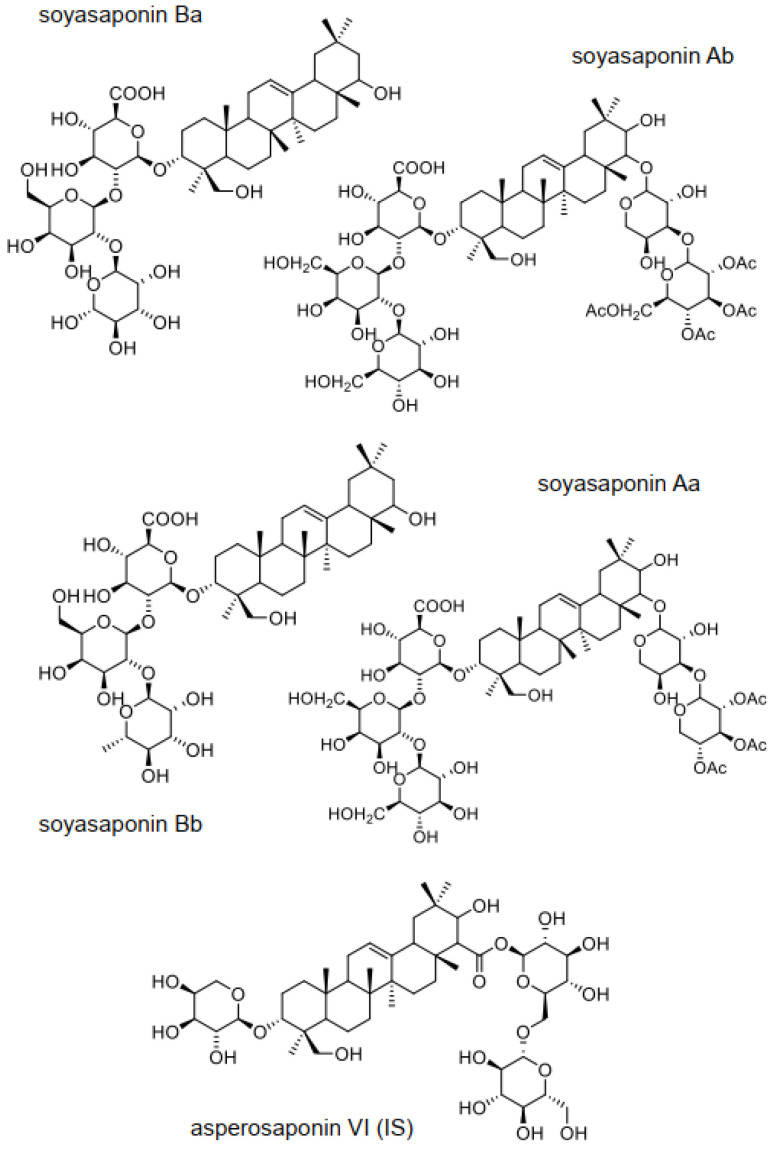
Chemical structures of soyasaponins (soyasaponin Aa, soyasaponin Ab, soyasaponin Ba, and soyasaponin Bb) quantified in this study and asperosaponin VI (used as internal standard (IS)).

Soyasaponins are amphiphilic compounds composed of polar sugar moieties attached to a nonpolar pentacyclic ring [15,16]. Soyasaponins are generally distributed between group A and B depending on the glycosylation positions of soyasapogenol A and soyasapogenol B [17,18]. Soyasaponin Aa and soyasaponin Ab are glycosylated at the C-3 and C-22 position of soyasapogenol A (group A), while soyasaponin Ba and soyasaponin Bb are glycosylated at the C-3 position of soyasapogenol B (group B). The structures of the studied soyasaponins are shown in Figure 1.

In addition to structural differences, group A soyasaponins contribute more to a bitter sensation than group B saponins [19], causing a major unpleasant taste of soybean-based dairy alternatives [20,21]. The group A soyasaponins are located both in soybean seed hypocotyls and cotyledons [3]. The removal of hypocotyls is usually performed during the production of soybean-based milk alternatives but is not enough to fully discard group A soyasaponins from the end products [3,4]. Hence, the residual soyasaponin concentration might still influence the bitterness of a product and thus limit the consumer acceptance.

Researchers have widely characterised the molecular structures of several forms of soyasaponins and have reported different methods for their quantification [4,22,23,24,25,26,27,28,29]. Indeed, saponin quantification is considered challenging due to the lack of chromophores in their molecular structure, leaving out the possibility of using UV light at a specific wavelength for quantification. Liquid chromatography (LC) coupled to electrospray ionisation mass spectrometry (ESI/MS) is an alternative approach providing significant selectivity and specificity without a need for the derivatization of analytes [16,25]. Despite extensive research carried out on different soybean foods, only a few studies have identified or relatively quantified the levels of soyasaponins in soybean-based dairy alternatives [4,29]. In the case of previously published methods, the liquid samples were initially pre-processed before extraction by being either freeze-dried [4] or dried by rotary evaporation [29]. The time-consuming application of these techniques may be considered the major drawback of previously reported quantification methods impeding the direct analysis of liquid samples. New extraction procedures are required to mitigate the issues with traditional analysis methods, allowing to save on equipment resources, increase the analysis throughput, and overall, facilitate the implementation of quality control throughout the development of new soybean-based dairy alternative products.

This study aimed to develop a selective extraction and quantification method for the determination of soyasaponins (soyasaponin Aa, soyasaponin Ab, soyasaponin Ba, and soyasaponin Bb) from a soybean-based yoghurt alternative matrix using hydrophilic interaction liquid chromatography with mass spectrometric detection (HILIC-MS). To our knowledge, there are no studies presenting the soyasaponin quantification method in which sample extraction has been performed directly from liquid soybean-based dairy alternative samples.

## 2. Materials and Methods

### 2.1. Food Samples

Soybean-based drink (SBD) and five soybean-based yoghurt alternatives (YA1, YA2, YA3, YA4, and YA5) from different producers were purchased from the local supermarket. Supplementary Table S1 provides nutritional and compositional information available on the label of the products. Samples were aliquoted and stored at −20 °C.

### 2.2. Chemicals and Materials

All solvents were HPLC grade and were purchased from Honeywell (Charlotte, NC, USA). Formic acid (FA) (98% for MS) and the ammonia solution (25% for LC-MS) were from Honeywell (Charlotte, NC, USA) and Merck KGaA (Darmstadt, Germany), respectively. The standard compounds soyasaponin Aa, soyasaponin Bb, soyasaponin Ba, and asperosaponin VI Phyproof^®^ Reference substances were from PhytoLab GmbH & Co. KG (Dutendorfer, Germany), and soyasaponin Ab was from MedChemExpress (Monmouth Junction, NJ, USA). Biotage Isolute^®^ PLD+ (100 mg/mL) cartridges were obtained from Biotage Sweden AB (Uppsala, Sweden). Ultrapure water (18.2 mΩ·cm) was prepared using MilliQ^®^ HX 7040SD equipped with MilliQ LC-Pak (Merck KGaA, Darmstadt, Germany).

### 2.3. Extraction Method for Samples

Soyasaponins were extracted according to the previously published method developed for pea and oat saponins [30] with some modifications. Briefly, the thawed homogeneous liquid sample was weighed (0.35–0.40 g) into a 5 mL volumetric flask (*n* = 3). Ultrapure water was added to the line and mixed thoroughly. The sample solution (native pH of yoghurt alternative was ~4.6) was alkalised to reach the sample pH 8 ± 0.25 using aqueous ammonia solution (5%, *v/v*) or aqueous FA (25%, *v/v*). Samples were incubated on a tube rotator Stuart SB3 ( Bibby Scientific Ltd, Staffordshire, UK) at room temperature for 30 min. After incubation, samples were centrifuged at 17,000 *g* at 10 °C for 10 min. After transferring the supernatant to a new Eppendorf tube, an equal volume of pure acetonitrile was added (MeCN, 1:1, *v:v*). The solution was mixed thoroughly and centrifuged at 14,800× *g* at 10 °C for 10 min to remove precipitated proteins. The supernatant (1000 µL) was passed through a PLD+ column using a vacuum manifold (VacMaster 10, Biotage Sweden AB, Uppsala, Sweden) at –0.5 bar. The filtrate (100 μL) was combined with an IS working solution (asperosaponin VI; 100 μL) and injected into the LC-MS.

### 2.4. Liquid Chromatography Mass Spectrometry

Analysis was performed as described previously [30] with adaptations to the analysis of soyasaponins. Briefly, a Waters UPLC^®^ system (Waters Corporation, Milford, MA, USA) attached to a Waters Quattro Premier XE Mass Spectrometer equipped with ZSpray™ Source was used to analyse the samples. The equipment was controlled by Waters MassLynx™ 4.1 (V4.1 SCN805, Waters Corporation, Milford, MA, USA). Mobile phase A consisted of ultrapure water containing 0.1% FA, and mobile phase B consisted of MeCN containing 0.1% FA. The gradient was changed as follows: 0–0.17 min at 10% A; 0.17–1.5 min linear gradient 10–70% A; 1.5–4.17 min at 70% A; 4.18 min switch to 10% A; 4.18–6.0 min at 10% A. The mobile phases were pumped at 200 µL/min flow rate. A BEH Amide column (1.0 × 50 mm, 1.7 μm) coupled with BEH Amide VanGuard Pre-column (2.1 × 5 mm) from Waters Corporation (Milford, MA, USA) were used to retain saponins. The autosampler and column heater were set at 8 °C and 50 °C, respectively.

The MS part of the method proposed for determination of oat and pea saponins [30] was adapted to target the quantification of soyasaponins. Based on a scan-type experiment of external standards, the deprotonated molecules [M-H]^−^ were chosen. The capillary voltage was set to −2.5 kV; cone voltages were optimised separately for every compound. The analysis was performed using negative electrospray ionisation (ESI^−^) mode using single-ion-recording (SIR) mass-to-charge ratios shown in Table 1. High-purity nitrogen was set as a cone and as desolvation gas at a rate of 25 L/h and 600 L/h, respectively. The temperature of the desolvation gas was set to 350 °C. Data acquisition and target analyte quantification were performed in Waters MassLynx™ and QuanLynx™ V4.1 (SCN805, Waters Corporation, Milford, MA, USA) and Microsoft Excel^®^ (Microsoft 365 Apps for enterprise, Microsoft Corporation, Richmond, WA, USA). Other parameters for MS were employed according to the description provided previously [30].

### 2.5. Calibration and Quantification

The stock solutions of soyasaponin Aa, soyasaponin Ab, and soyasaponin Bb (1000 mg/L) were prepared in ethanol (EtOH; 99.9% purity). The stock solution of soyasaponin Ba (1000 mg/L) was dissolved in ethanol:methanol solution (EtOH:MeOH; 1:1, *v/v*). The stock solution of asperosaponin VI used as IS (1000 mg/L) was prepared in ultrapure water. All solutions were aliquoted and stored at −80 °C. The working solution of asperosaponin VI (30 mg/L) was made freshly before the analysis using the aqueous MeCN (50%, *v/v*). The calibration curve standard solutions were diluted in MeCN:H_2_O:EtOH solution (50:36:14, *v/v*).

The IS (asperosaponin VI) working solution was added to the calibration curve standard solutions and the sample solutions before the injection, keeping the concentration of the IS constant. Calibration curve solutions were built for all soyasaponins and were run in triplicate (0.01–2.5 mg/L). Seven-point calibration curves of soyasaponins were prepared by plotting peak area ratios of soyasaponins/IS against the concentration of the external standard compound. The linear regression approach led to linear responses showing correlation coefficients of >0.99 for all analytes.

### 2.6. Validation of the Method

The European Medicines Agency (EMA) validation guideline was used to evaluate the following parameters during method validation: selectivity, specificity, calibration curve and range, limit of detection (LOD), limit of quantification (LOQ), precision, sample extraction recoveries, and matrix effect (ME) [31].

The calibration curve and range were evaluated via repeated measurements of standard solutions of soyasaponins consisting of eight individual points obtained from serial dilution of stock solutions. The LODs and LOQs were calculated using a previously published tutorial [32].

To determine the intra- and interday precision of the instrumental method, the standard solution and the IS were both injected six times and across three independent days to affirm the stability of the retention times (RTs) and peak areas. In addition, the repeatability (intraday) and intermediate precision (interday) of the whole method was investigated using YA2. Repeatability analysis was performed by six replicate analyses of samples on the same day. The intermediate precision of the method was by analysis of six replicates on three different days over four weeks under the same experimental conditions.

The total recoveries of analytes were assessed by spiking YA2 with a known amount of soyasaponins at four different concentration levels (unspiked, lower LOQ, middle LOQ, and upper LOQ) and performing the extraction methods as described above [33].

ME was evaluated by post-extraction spiking of sample extracts with calibration curve standard solutions and comparing the solvent-matched calibration curve slopes with matrix-matched slopes [32].

### 2.7. Statistical Analysis

Data analysis for sample extraction method development was performed in R 4.2.2 (The R Foundation for Statistical Computing, Vienna, Austria). ANOVA followed by Tukey–Kramer post hoc test was performed with R package ‘agricolae’ 1.3–5. The significance level was set to 0.05. The results are presented as mean with standard deviation (SD) or relative standard deviation (RSD). All analyses were repeated in triplicate if not marked otherwise.

## 3. Results and Discussion

### 3.1. Development of Liquid Chromatography Mass Spectrometry Method

**Figure 2:**
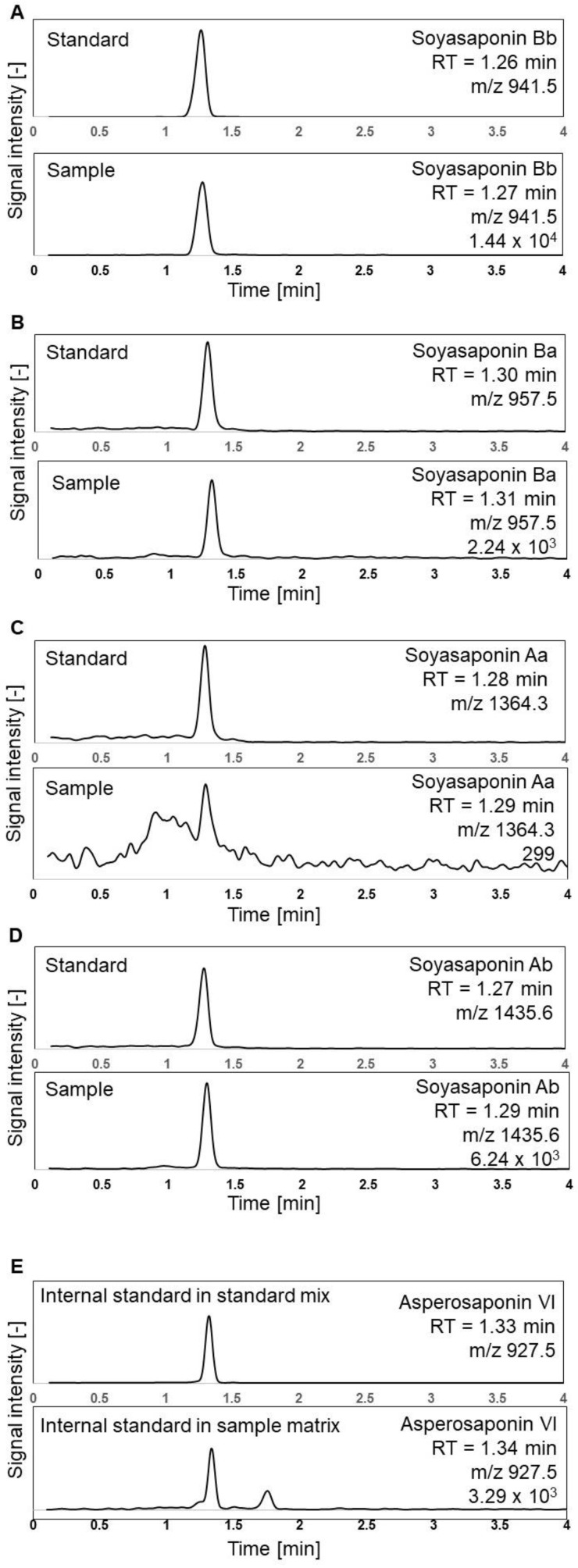
LC-MS chromatograms of external standard compounds and YA1 sample (SIR; ESI-): (**A**) soyasaponin Bb, (**B**) soyasaponin Ba, (**C**) soyasaponin Aa, (**D**) soyasaponin Ab, and (**E**) internal standard asperosaponin VI.

Previously reported LC-MS methods for the quantification of saponins varied from 6 to 80 min [2,30,34,35]. The shortest method with some modifications in the gradient and a total runtime of six minutes was used as a basis in our study. The SIR chromatograms shown in Figure 2 were obtained following an analysis of the soyasaponin standards, the sample of soybean-based yoghurt alternative, and the IS using the optimised analytical method described in Section 2.4. During the method development, we tested the multiple reaction monitoring (MRM) experimental conditions on our instrumentation. However, it did not enhance selectivity; instead, it notably decreased sensitivity by failing to generate consistent fragments. Nevertheless, the reasonably rapid retention of soyasaponins on the column and high-resolution peaks were achieved using the SIR mode. The proposed chromatographic method is more environmentally friendly and sustainable than previous approaches as it has a shorter duration, high-throughput nature, and reduced solvent usage.

Although soyasaponins include over one hundred different compounds [4], only the forms relevant to soybean-based yoghurt alternatives, including soyasaponin Bb, soyasaponin Ba, soyasaponin Aa, and soyasaponin Ab, were selected for total quantification. During method development, we also screened soybean-based yoghurt alternatives for the possible semi-quantification of the DDMP-conjugated form, but these compounds were not identified in the matrix. Plant-based yoghurt alternatives are typically pasteurised at 95 °C or undergo an ultra-high-temperature treatment above 100 °C during the production process, which helps to manage microbiological concerns and prolong the shelf life [1]. Under heat treatment, the thermo-sensitive DDMP conjugates of group B soyasaponins may degrade into non-DDMP saponin species. Hu et al. showed that the DDMP-conjugated B group saponins started to decrease already when heated at 65 °C [2]. Indeed, the range of possible analytes to quantitate could be potentially expanded by total synthesis or fractioning other soyasaponin compounds from raw materials, but in both cases, it is time-consuming, not cost-effective, and impractical for routine analysis in laboratories.

Asperosaponin VI was chosen as the IS for soyasaponins in this method based on its structural similarity to the triterpenoid core [36] (Figure 1) and LC-MS retention similar to the targeted analytes. Ideally, each soyasaponin target compound should be quantified using its corresponding isotopically labelled internal standard when these become more readily available, without the need for the custom total synthesis of standards or the cultivation of isotopically labelled soybeans.

### 3.2. The Influence of Sample pH on Saponin Extraction

A previously published method for the measurement of saponins in oat- and pea-based drinks [30] was used as a starting point for the development of an extraction method for soyasaponins from soybean-based yoghurt alternatives. Traditionally, saponin extraction is performed using ethanol or methanol from a solid fat-free sample before subsequent LC-MS analysis, the whole procedure starting from a Soxhlet-assisted fat-removing step, followed by the solvent extraction. The simplified procedure in this recently proposed method allowed the extraction of saponins from liquid samples with a minimal number of extraction steps and a small volume of solvents. The comprehensive comparison of the performance of this extraction method with selected traditional ones has been provided elsewhere [30].

In the present study, we focused on the exploration of the effect of the pH of the soybean-based yoghurt alternatives on the quantification of soyasaponins as the pH of these products is considerably lower than that of the SBD. The native pH of the SBD and the soybean-based yoghurt alternatives (YA1 and YA2) were 8.8 and 4.6–4.7, respectively. The effect of the native pH of the products and the effect of the pH adjustment before extraction on the yield of the extracted saponins are reported in Table 2. The SBD and the soybean-based yoghurt alternatives were analysed as described in Section 2.3: unspiked and spiked with all four soyasaponins and with or without a pH adjustment included in the sample extraction protocol. Indeed, in the samples at their native pH, the recoveries of soyasaponins in the SBD ranged from 80 to 109%, while for both yoghurt alternatives, the concentrations and recoveries were significantly lower than those observed in the SBD. Moreover, the recoveries of soyasaponins at native pH were similar among yoghurt alternatives. These results suggest that soyasaponin recoveries could be pH-dependent.

As the composition and nutritional information of the SBD, YA1, and YA2 were very similar (see Table S1), the following experiments were conducted with adjusted pHs of the samples to test the hypotheses of pH effects on soyasaponin recoveries (the results are shown in Table 2). The pH of the SBD was acidified to mimic the pH of the yoghurt alternatives, while the yoghurt alternatives were alkalised to mimic the pH of SBD. The experiment indicated that the SBD acidified to pH 4.2 had unacceptable recoveries of soyasaponins, ranging from 23 to 54%. On the contrary, yoghurt alternatives that were alkalised (pH 7.0 ± 0.2) resulted in higher soyasaponin recoveries: from 77 to 115% and from 83 to 98% in YA1 and YA2, respectively.

Based on these findings, additional experiments were conducted to assess the pH value at which soyasaponins would result in the highest and most meaningful recovery. YA2 was analysed at three additional pH values: 7.5 ± 0.2, 8.0 ± 0.2, and 8.5 ± 0.2. The results indicated that pH values of 7–8.5 had a beneficial influence on the recovery of soyasaponins, but there was no strict pH optimum value. ANOVA showed a statistical difference in the recoveries of soyasaponins at analysed pH values in most cases. The most acceptable recoveries were achieved at pH 7.5 ± 0.2 and pH 8.0 ± 0.2. Therefore, for further analyses, the method’s optimum pH value was chosen to be 8 ± 0.25.

Even though saponins are known as amphiphilic molecules, having a non-water soluble triterpene core and attached water-soluble sugar moieties, and are preferably soluble in organic solvents, soyasaponin Bb solubility is very low in the acidic region and increases drastically in the 6.5–7.3 pH region in aqueous buffers, having a solubility maximum in the range of 7 to 8 pH [37]. This fact elucidates the influence of different pH values of the samples on the soyasaponins recovery experiments. By adjusting the pH in soybean-based yogurt alternatives, the solubility issues of soyasaponins in acidic environments are overcome, enabling the direct analysis of liquid samples using a recently published method with modifications relevant to soyasaponins [30].

### 3.3. Validation of the Method

Validation was executed to assess the linear ranges, LODs and LOQs, precision, recoveries, and matrix effect of the proposed method for the determination of soyasaponins in yoghurt alternatives (Table 3). The calibration curves were constructed using a linear model with a weighing of 1/x. All four soyasaponins standards had high linearity (R^2^ > 0.99) in the 0.01–2.52 mg/L concentration range. The estimated LOQs for soyasaponins were ≤33.4 μg/L. The results of the LOQs were either lower or in accordance with previous research [34,35].

The repeatability of the method was investigated after the linearity of the soyasaponins was defined as acceptable. The results of the experiments are shown in the Supplementary Materials (Table S2). The RSDs of the peak RTs and the peak areas did not exceed 2% and 4%, respectively. It was observed that the intra- and interday RSDs for the whole method were lower than 12% and suitable for the routine analysis of soybean-based products. The precision observed using this method agreed with results reported by other LC-MS methods [34,35].

The recoveries of the soyasaponins were determined by spiking the YA2 with the analytes. Table 4 shows the results of the recovery of the YA2 at three spiking levels. The recoveries ranged from 81 to 101%. The obtained recoveries were acceptable according to the guidelines [31] and comparable with the previously published methods [2,30,34,35].

The experiment demonstrated that the soyasaponin Bb, soyasaponin Ba, soyasaponin Aa, and soyasaponin Ab MEs were 91%, 94%, 99%, and 94%, respectively. According to the guidelines, the achieved MEs were at an acceptable range [38], indicating sufficient sample clean-up.

According to validation guidelines, the method has confirmed sufficient validation performance regarding precision, recovery, sensitivity, and specificity. In addition, its efficiency and robustness for all the different yoghurt alternatives make the method valuable for screening and quality assurance.

### 3.4. Determined Concentrations of Soyasaponins in Soybean-Based Yoghurt Alternatives

We applied the developed and validated sample extraction method to quantify the soyasaponins in five soybean-based yoghurt alternatives (Table 5). All analysed samples had similar soyasaponin concentrations. The soyasaponin Bb, soyasaponin Ba, and soyasaponin Ab concentrations ranged from 11.7 to 14.5 mg/100 g, 2.6 to 4.2 mg/100 g, and 2.7 to 8.5 mg/100 g, respectively. The soyasaponin Aa concentration in all measured samples was below the LOQ.

Until now, limited data on the contents of soyasaponins in soybean-based dairy alternatives, including yoghurt alternatives, were available. A recently published study focused on characterizing the soyasaponin composition of 39 food products, including an analysis of 14 soybean-based milk alternative product [4]. The study showed that soyasaponin Aa was below the LOQ, soyasaponin Bb ranged from 27 to 308 mg/100 g dwb, soyasaponin Ba was quantified to be up to 14 mg/100 g dwb, and soyasaponin Ab ranged from 1 to 44 mg/100 g dwb in these products. Considering the average dry weight (~11%) of the samples in our study, our results showed that the average values of soyasaponin Bb, soyasaponin Ba, and soyasaponin Ab in the samples were 114 mg/100 g dwb, 29 mg/100 g dwb, and 54 mg/100 g dwb, which are in agreement with previously reported concentrations [4]. In another study, soyasaponin content was investigated [17], and the average sum of soyasaponin content in soybean-based milk alternatives was 39 μmol/100 g. The estimated average sum of quantified soyasaponins in the present study was 21 μmol/100 g in soybean-based yoghurt alternatives. Considering different methods used for quantification and possible different soy varieties, our results supported both previously published soyasaponin studies [4,17].

Previously, nine varieties of soybean were studied [3]. They found that only four varieties contained soyasaponin Aa (from 22.3 to 97.5 mg/100 g dwb of seed), two varieties contained soyasaponin Ab (from 75.8 to 95.5 mg/100 g dwb of seed), seven varieties contained soyasaponin Ba (up to 6.4 mg/100 g dwb of seed), and all nine studied varieties contained soyasaponin Bb (from 8.7 to 21.3 mg/100 g dwb of seed) among other soyasaponins. Generally, hypocotyls contained larger quantities of soyasaponins than cotyledons. Since hypocotyls might be removed during the production of soybean-based dairy alternatives, there may be smaller amounts of soyasaponins than in the original soybean seed [3]. The seeds contained DDMP-conjugated soyasaponin Bb and soyasaponin Ba, which might degrade into respective non-conjugated forms during the production of soybean-based yoghurt alternatives [1,2], resulting in higher soyasaponin contents reported in our study.

In another study, the soyasaponin content was analysed in tofu, one of the popular consumed soybean-based foods [4]. The authors showed that tofu had a quite diverse soyasaponin composition. Among others, tofu contained soyasaponin Aa at 80 mg/100 g dwb, soyasaponin Ab from 23 to 136 mg/100 g dwb, soyasaponin Ba from 5 to 11 mg/100 g dwb, and soyasaponin Bb ranging from 112 to 312 mg/100 g dwb of the product. The quantities of soyasaponins in tofu were also similar to the results obtained in the current study in the soybean-based yoghurt alternatives.

Although the number of analysed samples in the present study was small, due to the limited number of soybean-based yoghurt alternatives available on the local market, it was possible to demonstrate the applicability of the developed method on real samples. In the case of commercial end products, it is not possible to make assumptions about the content of saponins in the soybean varieties used for the production or the effectiveness of starter culture bacteria involved in the technological process to degrade saponins. Analysing the entire production chain, from soybean seeds to the final dairy alternative products, would provide a more comprehensive understanding of the mitigation of phytonutrients and allow for a thorough investigation of the entire technological process.

## 4. Conclusions

In this study, a new sample extraction method for the direct analysis of liquid samples was developed for the determination of soyasaponins in soybean-based yoghurt alternatives using HILIC-MS. The rapid LC-MS method was able to quantify soyasaponin Bb, soyasaponin Ba, soyasaponin Aa, and soyasaponin Ab using asperosaponin VI as an internal standard. The results show that the acidic pH of the soybean-based yoghurt alternatives significantly affected the quantification of soyasaponins, leading to unsatisfactory soyasaponin recoveries. To address this issue, the effect of alkalisation on the extraction yield of saponins was evaluated, and the highest yield (from 100 to 114%) was achieved at pH 8.0 ± 0.25. By adjusting the pH at the beginning of the sample extraction process, it became possible to achieve satisfactory recoveries of soyasaponins in soybean-based yoghurt alternatives. The developed method was validated using a soybean-based yoghurt alternative as a test matrix. Overall, the inter-day precision of the method was below 12%. This validated method could be applied in the analysis of commercially available soybean-based yoghurt alternatives and used in technology and product development, e.g., for the high-throughput screening of fermentation processes to unveil the saponins-degrading ability of starter cultures. The application of the presented method has the potential to enhance the acceptance of emerging and developed plant-based dairy alternatives by consumers by improving the quality of the final product and the taste by controlling the taste-active compounds. This method could also be extended for analyses of soyasaponins in dairy alternative products produced from other legume species.

## Figures and Tables

**Table 1:** The used m/z values and cone voltages for analytes.

Analyte	[M − H]^−^ *m/z* ± 0.5 Da	Cone Voltage (V)
Soyasaponin Bb	941.5	100
Soyasaponin Ba	957.3	120
Soyasaponin Aa	1364.3	120
Soyasaponin Ab	1435.6	120
Asperosaponin VI	927.5	120

**Table 2:** The relationship between sample pH and recovery (R) rates of soyasaponins during different extraction procedures. Samples: soybean-based drink (SBD); soybean-based yoghurt alternatives (YA1 and YA2). Each result represents mean ± standard deviation (*n* = 3). ANOVA statistical significance test was performed within sample matrices; means with different letters are significantly different at *p* < 0.05.

Sample	pH	Soyasaponin Bb		Soyasaponin Ba		Soyasaponin Aa		Soyasaponin Ab	
		mg/100 g	R ^1^ (%)	mg/100 g	R ^2^ (%)	mg/100 g	R ^3^ (%)	mg/100 g	R ^4^ (%)
SBD	8.8 (native)	12.6 ± 0.71 ^a^	98 ± 4 ^a^	2.43 ± 0.26 ^a^	109 ± 8 ^a^	<LOQ	85 ± 5 ^a^	<LOQ	80 ± 5 ^a^
	4.2 ± 0.2	1.2 ± 0.14 ^b^	23 ± 5 ^b^	0.82 ± 0.04 ^b^	26 ± 5 ^b^	<LOQ	54 ± 6 ^b^	<LOQ	51 ± 5 ^b^
YA1	4.7 (native)	0.84 ± 0.03 ^b^	20 ± 2 ^b^	0.56 ± 0.02 ^b^	25 ± 2 ^b^	<LOQ	43 ± 3 ^b^	0.58 ± 0.06 ^b^	41 ± 3 ^b^
	7.0 ± 0.2	5.39 ± 0.53 ^a^	77 ± 2 ^a^	1.03 ± 0.15 ^a^	109 ± 3 ^a^	<LOQ	104 ± 1 ^a^	1.82 ± 0.1 ^a^	115 ± 1 ^a^
YA2	4.6 (native)	1.3 ± 0.08 ^b^	27 ± 2 ^c^	0.54 ± 0.04 ^b^	25 ± 1 ^c^	<LOQ	48 ± 3 ^c^	3.07 ± 0.07 ^b^	63 ± 4 ^b^
	7.0 ± 0.2	10.94 ± 0.63 ^b^	85 ± 10 ^b^	2.36 ± 0.03 ^ab^	83 ± 8 ^b^	<LOQ	91 ± 7 ^ab^	8.09 ± 0.38b ^a^	98 ± 6 ^a^
	7.5 ± 0.2	13.63 ± 1.63 ^a^	89 ± 4 ^ab^	2.68 ± 0.06 ^a^	102 ± 5 ^ab^	<LOQ	86 ± 4 ^b^	9.93 ± 1.38 ^a^	103 ± 1 ^a^
	8.0 ± 0.2	14.43 ± 0.61 ^a^	100 ± 14 ^ab^	2.54 ± 0.34 ^a^	114 ± 16 ^a^	<LOQ	107 ± 15 ^a^	9.18 ± 0.36 ^a^	110 ± 15 ^a^
	8.5 ± 0.2	13.51 ± 1.97 ^a^	111 ± 1 ^a^	2.79 ± 0.4 ^a^	99 ± 8 ^ab^	<LOQ	74 ± 7 ^b^	9.01 ± 1.15 ^a^	91 ± 8 ^a^

^1^ soyasaponin Bb spike concentration: 2.10 mg/L. ^2^ soyasaponin Ba spike concentration: 1.86 mg/L. ^3^ soyasaponin Aa spike concentration: 1.91 mg/L. ^4^ soyasaponin Ab spike concentration: 2.03 mg/L.

**Table 3:** The linear range, calibration curve, limits of detection (LODs), and limits of quantification (LOQs) of soyasaponins.

Analyte	Linear Range (mg/L)	Calibration Curve	R^2^	LOD (μg/L)	LOQ (μg/L)
Soyasaponin Bb	0.01–2.52	y = 0.7699x + 0.0048	0.9930	0.2	12.6
Soyasaponin Ba	0.02–2.26	y = 0.2949x + 0.0025	0.9975	8.0	33.4
Soyasaponin Aa	0.02–2.33	y = 0.3994x + 0.0021	0.9965	7.0	27.0
Soyasaponin Ab	0.01– 2.48	y = 0.3259x + 0.0033	0.9943	1.4	25.1

**Table 4:** The recoveries of soyasaponins in soybean-based yoghurt alternative matrix (mean ± standard deviation (*n* = 3)).

Spiking Level	Soyasaponin Bb ^1^		Soyasaponin Ba ^2^		Soyasaponin Aa ^3^		Soyasaponin Ab ^4^	
	mg/L	R, %	mg/L	R, %	mg/L	R, %	mg/L	R, %
L1	0.03	95 ± 4	0.02	97 ± 5	0.02	101 ± 9	0.03	96 ± 3
L2	0.77	87 ± 4	0.68	87 ± 3	0.62	81 ± 1	0.8	90 ± 2
L3	1.54	82 ± 2	1.36	86 ± 2	1.23	81 ± 0	1.61	88 ± 0

^1^ Unspiked matrix soyasaponin Bb concentration: 0.99 mg/L. ^2^ Unspiked matrix soyasaponin Ba concentration: 0.27 mg/L. ^3^ Unspiked matrix soyasaponin Aa concentration: 0.03 mg/L. ^4^ Unspiked matrix soyasaponin Ab concentration: 0.72 mg/L.

**Table 5:** Soyasaponins content (mg/100 g) in soybean-based yoghurt alternatives (mean ± standard deviation (*n* = 3)). ANOVA statistical significance test was performed across all analysed samples; means with different letters are significantly different at *p* < 0.05.

mg/100 g				
Sample Code	Soyasaponin Bb	Soyasaponin Ba	Soyasaponin Aa	Soyasaponin Ab
YA1	14.5 ± 0.4 ^a^	2.8 ± 0.2 ^b^	<LOQ	3.5 ± 0.4 ^bc^
YA2	11.9 ± 0.8 ^b^	2.9 ± 0.2 ^b^	<LOQ	7.7 ± 0.3 ^a^
YA3	12.6 ± 0.5 ^b^	4.0 ± 0.4 ^a^	<LOQ	8.5 ± 0.4 ^a^
YA4	11.7 ± 0.4 ^b^	2.6 ± 0.1 ^b^	<LOQ	2.7 ± 0.3 ^c^
YA5	13.3 ± 1.0 ^ab^	4.2 ± 0.6 ^a^	<LOQ	4.5 ± 0.5 ^b^

## Data Availability

The data are contained within the article or the Supplementary Materials.

## Supplementary Materials

The following supporting information can be downloaded at https://www.mdpi.com/article/10.3390/foods12112164/s1. Table S1: Nutritional information of analysed products. Table S2: Repeatability of retention times (RTs), peak areas of soyasaponins, and precision of the whole method.
