# Supplementary material for: Development of Extraction Method for Determination of Saponins in Soybean-Based Yoghurt Alternatives: Effect of Sample pH
Source: Foods. 2023 May 27;12(11):2164. doi: 10.3390/foods12112164 (PMC10252951; doi:10.3390/foods12112164)
Supplement: Supplementary file 1 [file foods-12-02164-s001.zip › foods-2346325-supplementary.pdf]

Supplementary Information

Development of extraction method for determination of saponins in soybean-based yoghurt alternatives: effect of sample pH

Anastassia Bljahhina[1,2], Maria Kuhtinskaja[2] and Tiina Kriščiunaite[1*]

[1]Center of Food and Fermentation Technologies (TFTAK), Mäealuse 2/4, 12618 Tallinn, Estonia

[2]Department of Chemistry and Biotechnology, Tallinn University of Technology, Akadeemia tee 15, 12618 Tallinn, Estonia

*Corresponding: tiina@tftak.eu

Table S1: Nutritional information of analysed products.

Sample code	Producer[1]	information
**YA1**	A	Ingredients: Soya base (Water, Hulled soya beans (10.7%)), Sugar, Calcium (Tri-calcium citrate), Stabiliser (Pectins), Acidity regulators (Sodium citrates, Citric acid), Flavouring, Sea salt, Antioxidants (Tocopherol-rich extract, Fatty acid esters of ascorbic acid), Vitamins (B12, D2), Yogurt cultures (S. thermophilus, L. bulgaricus).<br>Nutritional values per 100 g: Energy: 212 kJ / 51 kcal;<br>Fat: 2.3 g; of which saturates: 0.4 g; mono-unsaturates: 0.5 g; polyunsaturates: 1.4 g; Carbohydrate: 2.1 g; of which sugars: 2.1 g; Fibre: 1 g; Protein: 4 g; Salt: 0.25 g; Vitamin D: 0.75 µg; Vitamin B12: 0.38 µg; Calcium: 120 mg
**YA2**	B	Ingredients: Water, organic soy (11%), organic corn starch, salt, yogurt cultures<br>Nutritional values per 100 g: Energy: 209 kJ / 50 kcal; Fat: 2,6 g; of which saturated fat: 0,4 g; Carbohydrate: 1,5 g; of which sugars: 0,4 g; Protein: 47 g; Salt: 0,07 g
**YA3**	C	Ingredients: Soy drink 76.5% (water, soybeans 8.7%), sugar, peaches (7.9%), water, maracuja (0.9%), modified starch, dextrose, calcium phosphate, thickener: pectin, citrus fiber, flavorings, acidity corrector: tartaric acid, vitamins: Riboflavin (B2), B12, D2, live cultures.<br>Nutritional values per 100 g: Energy: 320 kJ, 76 kcal; Fat: 1.5 g; of which saturates: 0.2 g; mono-unsaturates: 0.2 g; polyunsaturates: 1.1 g; Carbohydrate: 12 g; of which sugars: 11 g; Fibre: 0.7 g; Protein: 3.2 g; Salt: 0.01 g; Vitamin B2: 0.21 mg; Vitamin B12: 0.38 µg; Vitamin D2: 1.5 µg; Calcium: 120 mg
**YA4**	A	Ingredients: Soya base (Water, Hulled soya beans (9.7%)), Peach (10.9%), Sugar, Acidity regulators (Citric acid, Sodium citrates), Stabiliser (Pectins), Tri-calcium phosphate, Flavouring, Sea salt, Antioxidants (Tocopherol-rich extract, Fatty acid esters of ascorbic acid), Carrot extract, Vitamins (B2, B12, D2), Yogurt cultures (S. thermophilus, L. bulgaricus).<br>Nutritional values per 100 g: Energy: 292 kJ / 69 kcal; Fat: 2.1 g; of which saturates 0.4 g; mono-unsaturates: 0.5 g; polyunsaturates: 1.2 g; Carbohydrate: 8.3 g; of which sugars: 8.2 g; Fibre: 0.9 g; Protein: 3.6 g; Salt: 0.25 g; Vitamin D: 0.75 µg; Vitamin B2: 0.21 mg; Vitamin B12: 0.38 µg; Calcium: 125 mg
**YA5**	A	Ingredients: Soya base (Water, Hulled soya beans (15.7%)), Sugar, Stabiliser (Pectins), Calcium (Tri-calciumcitrate), Acidity regulators (Sodium citrates, Citric acid), Natural flavouring, Sea salt, Antioxidants (Tocopherol-rich extract, Fatty acid esters of ascorbic acid), Vitamins (B12, D2), Yogurt cultures (S. thermophilus, L. bulgaricus).<br>Nutritional values per 100 g: Energy: 283 kJ / 68 kcal; Fat: 3.3 g; of which saturates: 0.6 g; mono-unsaturates: 0.7 g; polyunsaturates: 2 g; Carbohydrate: 2.6 g; of which sugars: 2.5 g; Fibre: 1.5 g; Protein: 5.8 g; Salt: 0.36 g; Vitamin D: 0.75 µg; Vitamin E: 1.3 mg; Vitamin B12: 0.38 µg; Calcium: 120 mg
**SBD**	D	Ingredients: soya base 99% (water, soya beans 7,5%), calcium carbonate, stabiliser: gellan gum; acid regulator: potassium carbonat; vitamine: D, vitamine, natural flavouring, salt<br>Nutritional values per 100 ml: Energy 144kJ / 35 kcal; Fat: 1.9 g of which saturates 0.3 g; Carbohydrate: 0.8 g of which sugars 0.7 g; Fibre: 0.5 g; Protein 3.3 g; salt 0.11 g; Calcium: 120 mg; vitamin D2 0.75 µg; vitamin B12 0.38 µg

[1] different letters mark different producers

Table S2. Repeatability of retention times (RTs), peak areas of soyasaponins and precision of the whole method

analyte	m/z	RT (min)	RT RSD (%)		Peak area RSD (%)		Precision[1] RSD (%)	
			intra-day (n = 6)	inter-day (n = 18)	intra-day (n = 6)	inter-day (n = 3)	intra-day (n = 6)	inter-day (n = 3)
Soyasaponin Bb	941.5	1.28	0.03	0.7	1.33	1.1	6	11
Soyasaponin Ba	957.3	1.32	0.70	0.8	1.73	2.0	5	12
Soyasaponin Aa	1364.3	1.30	0.16	0.5	0.96	2.2	*	*
Soyasaponin Ab	1435.6	1.28	0.37	0.6	0.19	3.5	4	12
Asperosaponin VI	927.5	1.34	0.10	1.5	3.88	3.0	-	-

[1] precision of the whole method
